# *In vivo* Two-Photon Imaging of Anesthesia-Specific Alterations in Microglial Surveillance and Photodamage-Directed Motility in Mouse Cortex

**DOI:** 10.3389/fnins.2019.00421

**Published:** 2019-05-07

**Authors:** Weilun Sun, Kunimichi Suzuki, Dmytro Toptunov, Stoyan Stoyanov, Michisuke Yuzaki, Leonard Khiroug, Alexander Dityatev

**Affiliations:** ^1^Molecular Neuroplasticity, German Center for Neurodegenerative Diseases (DZNE), Magdeburg, Germany; ^2^Department of Neurophysiology, Keio University School of Medicine, Tokyo, Japan; ^3^Neurotar Ltd., Helsinki, Finland; ^4^HiLIFE/Neuroscience Center, University of Helsinki, Helsinki, Finland; ^5^Medical Faculty, Otto-von-Guericke University, Magdeburg, Germany; ^6^Center for Behavioral Brain Sciences, Magdeburg, Germany

**Keywords:** microglia, motility, two-photon imaging, anesthesia, isoflurane, ketamine

## Abstract

Two-photon imaging of fluorescently labeled microglia *in vivo* provides a direct approach to measure motility of microglial processes as a readout of microglial function that is crucial in the context of neurodegenerative diseases, as well as to understand the neuroinflammatory response to implanted substrates and brain-computer interfaces. In this longitudinal study, we quantified surveilling and photodamage-directed microglial processes motility in both acute and chronic cranial window preparations and compared the motility under isoflurane and ketamine anesthesia to an awake condition in the same animal. The isoflurane anesthesia increased the length of surveilling microglial processes in both acute and chronic preparations, while ketamine increased the number of microglial branches in acute preparation only. In chronic (but not acute) preparation, the extension of microglial processes toward the laser-ablated microglial cell was faster under isoflurane (but not ketamine) anesthesia than in awake mice, indicating distinct effects of anesthetics and of preparation type. These data reveal potentiating effects of isoflurane on microglial response to damage, and provide a framework for comparison and optimal selection of experimental conditions for quantitative analysis of microglial function using two-photon microscopy *in vivo*.

## Introduction

Microglia are highly dynamic immune cells of the central nervous system which have a wide range of functions and play important roles in brain physiology and pathologies. Previous studies have shown microglia make contacts with the vasculature, neurons, and astrocytes (Nimmerjahn et al., 2005) and may promote dendritic spine formation (Parkhurst et al., 2013; Miyamoto et al., 2016). The homeostatic activity of microglia involves protecting the brain from a barrage of frequently occurring small lesions, ranging from ruptured micro-capillaries to dying cells releasing toxic content into tightly controlled extracellular space (Kraft and Harry, 2011; Nayak et al., 2014). This protective function relies on at least two distinct types of motility of microglial processes: (i) surveilling motility, and (ii) damage-directed motility.

The surveilling, a.k.a. resting, motility refers to surveillance of the microglial cell's immediate environment, which enables timely detection of brain damage and pathogen infiltration (Hanisch and Kettenmann, 2007). This surveilling motility crucially depends on the membrane potential and requires the constitutive hyperpolarizing activity of a specific type of inwardly-rectifying potassium channel called TWIK-related Halothane-Inhibited K^+^ channel, THIK-1 (Madry et al., 2018). Resting motility also underlies microglia-synapse interactions, during which microglial processes monitor synaptic function and modify the “wiring” of neuronal circuits by strengthening or eliminating individual synapses (Wake et al., 2009; Tremblay et al., 2010; Schafer et al., 2012). Noteworthy, microglial surveillance can be selective/directional in the sense that microglial cells monitor and interact with neurons during conditions of cerebral calcium reduction in the normal and diseased brain (Eyo et al., 2015).

The damage-directed motility manifests as an extension, or protrusion, of microglial processes toward a micro-lesion site (Davalos et al., 2005; Nimmerjahn et al., 2005; Haynes et al., 2006; Gyoneva et al., 2014). In contrast to surveillance, damage-directed motility of microglial processes does not require THIK-1 activity and hyperpolarized membrane potential but depends on activation of the metabotropic purinergic receptor P2Y12 by ATP released from the damaged cell(s) (Haynes et al., 2006). Other types of purinergic receptors, including P2Y6, are involved in the phagocytic activity of microglia at the site of damage (Hidetoshi et al., 2012). Microglial response to brain damage is also regulated by TAM (Tyro3, Axl, and Mer) receptors (Fourgeaud et al., 2016). These receptors play multiple functional roles as homeostatic regulators in adult tissues and organ systems that are subject to continuous challenge and renewal throughout life (Lemke, 2013) as well as in nervous system development and diseases (Pierce and Keating, 2014).

General anesthesia is commonly used during *in vivo* imaging, however, it may dramatically alter the function of brain cells, including microglia. Known effects of anesthetics range from suppressing neuronal firing and changing inter-cortical synchronization to altering the morphology of glial processes and affecting turnover rates of dendritic spines (Li et al., 2010; Huh and Cho, 2013; Pryazhnikov et al., 2018). Recently, Madry et al. (2018) have demonstrated that isoflurane and related gaseous anesthetics strongly suppressed the motility of microglial processes in acute brain slices. This suppression was mediated by anesthetics' blocking effect on THIK-1. The authors demonstrated that both types of motility, non-directional and damage-directed, were suppressed when isoflurane was applied either *in vivo* prior to slice preparation or *in situ* by direct exposure of slices.

Interestingly, suppression of microglial motility appears to be restricted to gaseous anesthetics, because injectable anesthetics such as urethane did not have such an effect (Madry et al., 2018). It remains unknown, however, whether another injectable anesthetic ketamine, which is widely used in mouse imaging experiments, also affects non-directional and/or damage-directed motility of microglial processes. Furthermore, since the motility-suppressing effect of isoflurane was observed *ex vivo* by imaging in brain slices, the question of whether this effect also occurs *in vivo* remains unanswered and must be addressed using microscopic analysis of the living brain. Besides anesthesia, other factors can affect the outcome of *in vivo* experiments, such as the interval between implantation of a cranial window and imaging (hours for acute preparation vs. weeks for chronic preparation).

Here, we set out to address these issues by using *in vivo* two-photon microscopy and directly compared the kinetics of both resting and damage-directed motility of microglial processes between awake and anesthetized states in acute and chronic preparations.

## Materials and Methods

### Animals

Seven 3–4 month-old male CX3CR1-GFP heterozygous C57BL/6JCRL mice (B6.129P-Cx3cr1tm1Litt/J) (Jung et al., 2000) (Jackson Laboratory; Stock No.005582) bred at the DZNE (Magdeburg) animal facility were used in this study. Since we were comparing different conditions in the same animal, no randomization was performed. The mice were housed individually under a fixed 12-h light/dark cycle with food and water available *ad libitum*. All animals were treated in strict accordance with ethical animal research standards defined by the German law and approved by the Ethical Committee on Animal Health and Care of Saxony-Anhalt state, Germany (license number: 42502-2-1346).

### Surgical Procedures

Surgeries were performed as described previously (Senkov et al., 2006; Holtmaat et al., 2009). Briefly, mice were sedated in a chamber with isoflurane (Baxter, Germany) prior to being stabilized in the stereotaxic apparatus (SR-6M, Narishige Scientific Instrument Lab, Japan). Under stereotaxic head fixation, isoflurane level was adjusted by using an isoflurane vaporizer (Matrx VIP 3000, Midmark, USA) to 4% for induction and 1.5–2% during surgery with 0.4 L/min O_2_, while the depth of anesthesia was monitored by breathing rate. During surgery, mice were placed on a heating blanket connected to a temperature controller (ATC1000, World Precision Instruments, USA) with the temperature maintained at 37°C. An intraperitoneal (i.p.) injection of ketoprofen (5 mg/kg, body weight) was done before surgery to prevent inflammation and pain. In order to protect eyes from dehydration and irritation, ophthalmic ointment was applied. After shaving the hair and cleaning the scalp with 70% ethanol, a cutaneous incision was made and a flap of skin, ~ 1 cm^2^, covering the skull over both hemispheres was removed. Then the surface of the skull was cleaned with 10% povidone-iodine (Dynarex) and 3% hydrogen peroxide solution (Sigma-Aldrich, Germany). A craniotomy (diameter ~5 mm, centered over midline) was gently performed with a high-speed dental drill (Eickemeyer, Germany) over the cortical area between bregma and lambda. To prevent heat-induced damage of the underlying cortex, drilling was interrupted, and sterile saline was applied on the skull periodically. The craniotomy was then covered with a circular glass coverslip (6 mm diameter, Thermo Fisher Scientific, Germany) and fixed with cyano-acrylic glue (Roti Coll-1, CarlRoth, Germany). A custom-built 3D printed metal head-plate (i.materialize, Belgium) was then implanted on the exposed skull with glue and cemented with dental acrylic (Paladur, Heraeus Kulzer, Germany). After all procedures, mice were kept in a home cage with red-lamp warming for ~30 min until they recovered from anesthesia.

### Two-Photon *in vivo* Imaging

**Figure 1:**
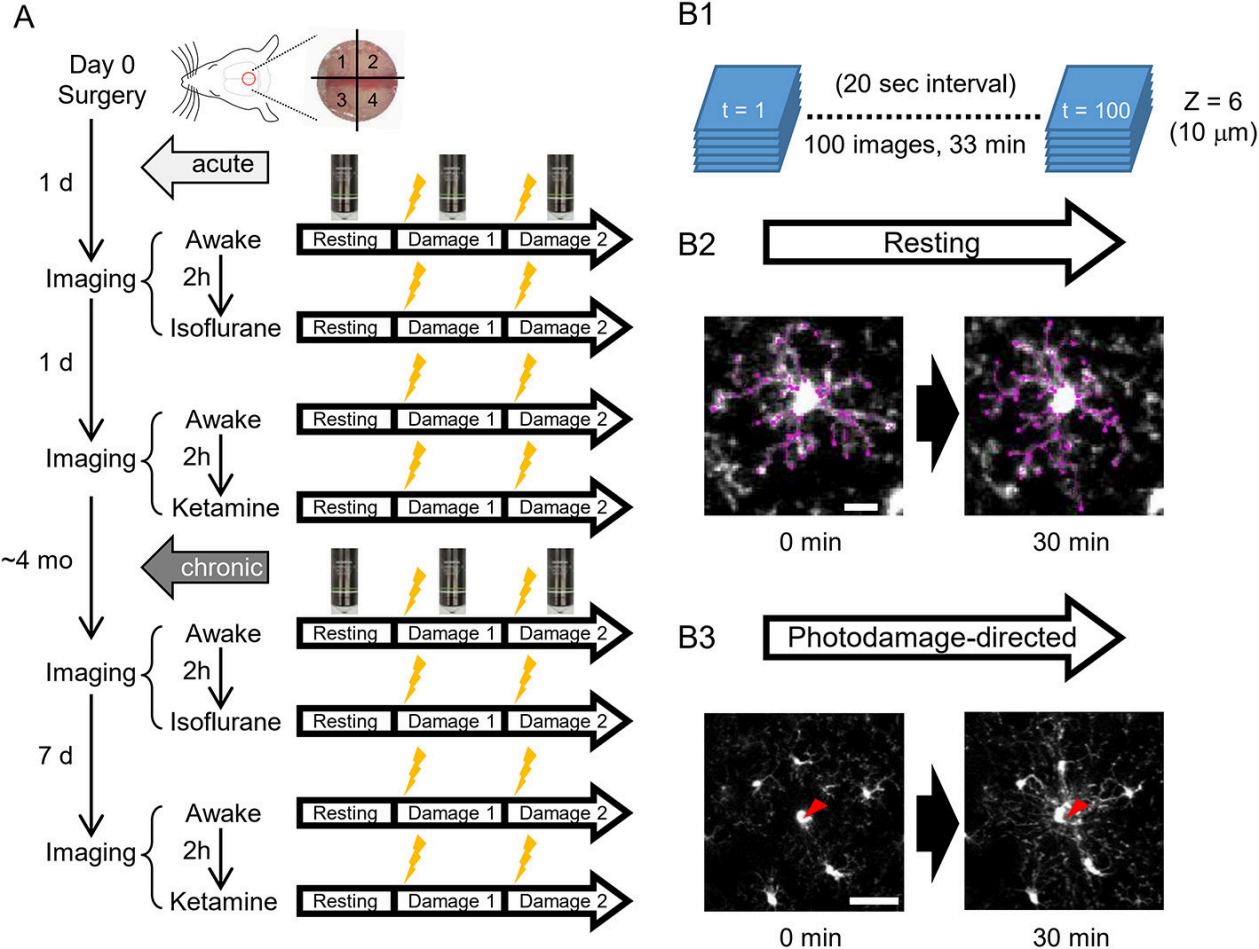
A scheme of experiments. **(A)** Schedules of *in vivo* two-photon imaging of microglia in awake, isoflurane- and ketamine-anesthetized mice. For each condition, imaging is done in a resting and photodamage state. The same imaging protocols are applied acutely, i.e., 1–2 days after the surgery (5 animals), and/or in chronically implanted mice (around 4 months after the surgery, 4 animals). **(B)** Imaging examples in the resting and photodamage conditions. **(B1)** Z-stacks (6 images) are acquired at a 20 s interval for 33 min, generating 100 time frames. **(B2)** Two-dimensional tracing of microglia processes with 30 min interval in the resting state (scale bar, 10 μm). **(B3)** Analysis of microglia response to photodamage. A red arrowhead indicates the center of damage induced by the high-power laser stimulation (scale bar, 50 μm).

To address the differences of microglial morphology and dynamics between awake and anesthetized conditions, we performed the same imaging procedures in awake, isoflurane- and ketamine-anesthetized conditions, respectively. Because we were using the same animals for different conditions and the anesthesia method was too transparent, we could not perform blinding in this study. GFP-labeled microglial cells were imaged by a custom-built two-photon microscope (Thorlabs, USA) with a Ti: Sapphire pulsing laser (Chameleon, Coherent, USA) tuned to 850 nm. A 20 X water immersion lens (1.00 N.A.; Olympus, Japan) at a zoom of 1.0 was used to acquire 512 × 512 pixels images with a field-of-view of 393 × 393 μm throughout all imaging sessions. Imaging and two-photon laser ablation protocols were modified from previous studies (Davalos et al., 2005; Nimmerjahn et al., 2005) and time-plan of experiments is shown in Figure 1A. In order to avoid the effects of imaging under one condition on subsequent imaging under another condition, hemispheres were divided into quadrants and distinct quadrants were used in different imaging sessions (as shown in Figure 1A). For the first day after surgery, quadrants 1 and 3 were used for awake condition and isoflurane-anesthetized condition, respectively. Quadrants 2 and 4 were used for second awake condition and ketamine-anesthetized condition 2 days after surgery, respectively. 1&3 (anterior) and 2&4 (posterior) quadrants were counterbalanced between animals. For awake-condition (1 day after surgery), mice were head-fixed under the two-photon microscope using a Mobile HomeCage device (Neurotar, Finland) in which head-fixed animals could freely move a light cage around them. After 10 min habituation in the Mobile HomeCage, time-lapse imaging was performed for 33 min (100 frames, 20 s interval) by recording z-stacks (6 optical sections with a step size of 2 μm) around 100–150 μm below the pial surface to monitor microglial morphology and dynamics in the resting state. After acquiring images in the resting state, a single cell laser ablation was achieved by focusing a two-photon laser beam at a single microglia cell in the superficial layer of the cortex to induce a highly precise and reproducible local injury. The wavelength of the pulsed infrared laser was set to 780 nm and the power was ~150–200 mW at the target. The laser beam was parked at the targeted cell by zooming in to an 18 × 18 μm field-of-view for ~0.5 s to create a small highly localized photo-damage. The final injury area was determined by a relatively bright autofluorescent sphere (~ 20 μm diameter) around the focal point. Immediately after the single cell laser ablation, the same imaging procedure as in the resting state was taken. Afterward, another laser ablation was performed at least 400 μm away from the first one. After imaging in awake mice, 2-h rest was given to each animal before we started the imaging session in the isoflurane-anesthetized condition. The same procedures of imaging as in awake-condition were applied in the other hemisphere, but the animal was head-fixed using a custom-made frame with a heating pad to keep body temperature constant, and anesthetized by 1.5% isoflurane during the imaging, which started 10 min after anesthesia induction, i.e., with the same interval as the habituation period in awake condition. On the next day (2 days after surgery), we repeated awake-condition imaging in another quadrant as described above. After 2-h rest, animals were intraperitoneally injected with ketamine (90 mg/kg body weight) and xylazine (18 mg/kg body weight) in 0.9% NaCl solution and placed in a head fixation frame in the same manner as used for imaging in isoflurane-anesthetized condition. During the imaging session, ketamine was added approximately every 35 min to maintain anesthesia between basal recording and after-damage recordings. The imaging procedure in ketamine-anesthetized condition was the same as for the other two conditions. All imaging sessions under different conditions were repeated at 4 months post-surgery, i.e., after the cranial window reached a chronic (steady-state) condition and the only difference was the interval between awake 1 and awake 2 imaging sessions, which was 7 days instead of 1 day (Figure 1A).

### Analysis of Microglia Dynamics in Surveillance and Photodamage-Directed State

Before the quantitative analysis, the movement artifacts in obtained images (100 frames and 6 z-stacks) were neutralized using ImageJ software (Schindelin et al., 2012). Six sequential z-stacks of images were first subjected to the ImageJ plug-in StackReg/TurboReg with “rigid body” mode and the aligned z-stacks were maximally projected to a single image at every frame (Thévenaz et al., 1998). The projected images aligned along z-axis were then subjected to the same alignment processing along the time-axis (100 frames). Briefly, every 10 frames were averaged to improve the signal-to-noise ratio and compared between the first 10 frames (0–3 min) and the last 10 frames (30–33 min). Each microglia process was 2-dimensionally traced by the Fiji plug-in Simple Neurite Tracer (Longair et al., 2011). Tracing of microglial processes was done by an experimenter blind to the experimental conditions. The longest processes stemming from the microglial cell body were traced as the primary processes, and the arborized processes from each primary process were traced to include the secondary, but not tertiary or further, branches. We calculated the average length and number of primary processes per microglia, the average length of arborized primary processes per microglia, the average terminal number in the arborized primary processes, the total length of the primary + secondary (defined as “all”) processes and the total numbers of the all processes per microglia. Each process was traced twice, with 30 min interval, and the changes were qualified as “Disappear,” “Decrease,” “No change,” “Increase” and “Appear” with respect to each primary process, arborized process, and all processes. The parameters were measured and averaged from at least 3 microglial cells per animal. Our quantification method is similarly based on the manual tracing of processes and provides the terminal number that is comparable to the previously reported value by other researchers (on average, 18 vs. 20 in control conditions; Nimmerjahn et al., 2005). Although our measurements resulted in a shorter total length of processes than previously reported (226 vs. 400 μm; Nimmerjahn et al., 2005), probably due to differences in the imaging conditions and the criteria used for tracing of processes, we can compare the lengths of processes among different conditions within our study. The dynamics of microglial processes is similar for our and the previous work (same fractions of processes which were increased or decreased, resulting in constant total length), suggesting the validity of our measurements.

For the analysis of damage-directed response, the quantification of microglial process velocity was based on the intensity of microglia around damage spot, which is a method similar to Davalos et al. (2005) and rendered the comparable value (1.6 vs. 1.2 μm/min) in control conditions. In detail, as a method to calculate the velocity of damage-directed process motility, the center of each ROI was set to the microglial processes apparent attraction point. Using the Radial Profile Extended ImageJ plugin, the normalized intensity profiles for R ≤ 75 μm for each time point were calculated. A set of these 100 radial profiles was converted into grayscale images and 3D height images using a custom-built R script. Microglial processes were readily visible as a distinct stripe on the 2D images and as a ridge on the 3D images. Using the 2D image, a line ROI was drawn along the path of the microglial processes and their projections onto time (Δt) and distance to the damage (ΔD) axes. *Velocity was measured as ΔD*/Δ*t*. Additionally, in order to get a better visualization of the events happening during the microglial activity, a stack was converted into a color-coded image, where each time point was coded with its own color (**Figure 5A**). Since the velocity of damage-directed process motility is a crucial parameter in such studies, we additionally applied another—more objective—method by using the Sholl analysis applied to the aligned z-projection images (100 frames) to measure the advance of microglia process toward the injury spot. The measurement was done from the time when the microglia process started to migrate toward the injury center to the time when the microglia reached the injury spot. To reduce the noise and background and specifically detect the dynamics of the microglia process, the Gaussian filter (σ = 0.5) and background subtraction (10 px) was applied to every image and the bright signals of microglia soma and injury spot artifact were deleted by the masked images derived from Analyze Particles calculation (>50 px, default threshold, at the first frame). Then the process frontier was measured by detecting the peak intensity of microglia processes on the circumference at the distance from the injury spot. The auxiliary line was drawn to enhance the visible frontier in some images with a lower signal-to-noise ratio. The distance of peak intensity was plotted along the time and the regression curve was calculated as a linear line with the high correlation coefficient between the observed and predicted values (*R*^2^ > 0.9). The slope was used as the calculated velocity (Supplementary Figure 3A). Finally, we defined the injury size as the size of the relatively bright autofluorescent sphere around the focal point immediately after laser ablation and the activation area was measured according to the radius of the apparent microglia process frontier starting to approach the center of the photodamage (Supplementary Figure 4). Statistical analysis was performed using XLSTAT (STATCON, Germany), as described in Results.

## Results

To analyze microglia functions *in vivo*, we implanted a glass-covered cranial window above the cortical region between bregma and lambda, including the retrosplenial cortex, which is one of the cortical areas critically involved in spatial navigation that is affected at early stages of Alzheimer's disease. Two-photon imaging was performed either in acute preparation, i.e., on the first and second days after the window implantation (5 mice), or in chronic preparation, i.e., 4 months after the implantation (3 of the 5 acutely imaged mice and 1 new mouse) (Figure 1). The effects of two commonly used anesthetics, isoflurane and ketamine, were analyzed. The longitudinal design allowed a within-animal comparison of microglial morphology and motility in awake vs. anesthetized conditions on the same day, with a 2-h interval between sessions. Each session included imaging of microglia at the resting state and after photodamage induced at two spatially separated sites by laser ablation of a single microglia cell (Figure 1), as the only unequivocally defined target in our experimental settings.

### Analysis of Structure and Motility of Microglia at the Surveilling State

**Figure 2:**
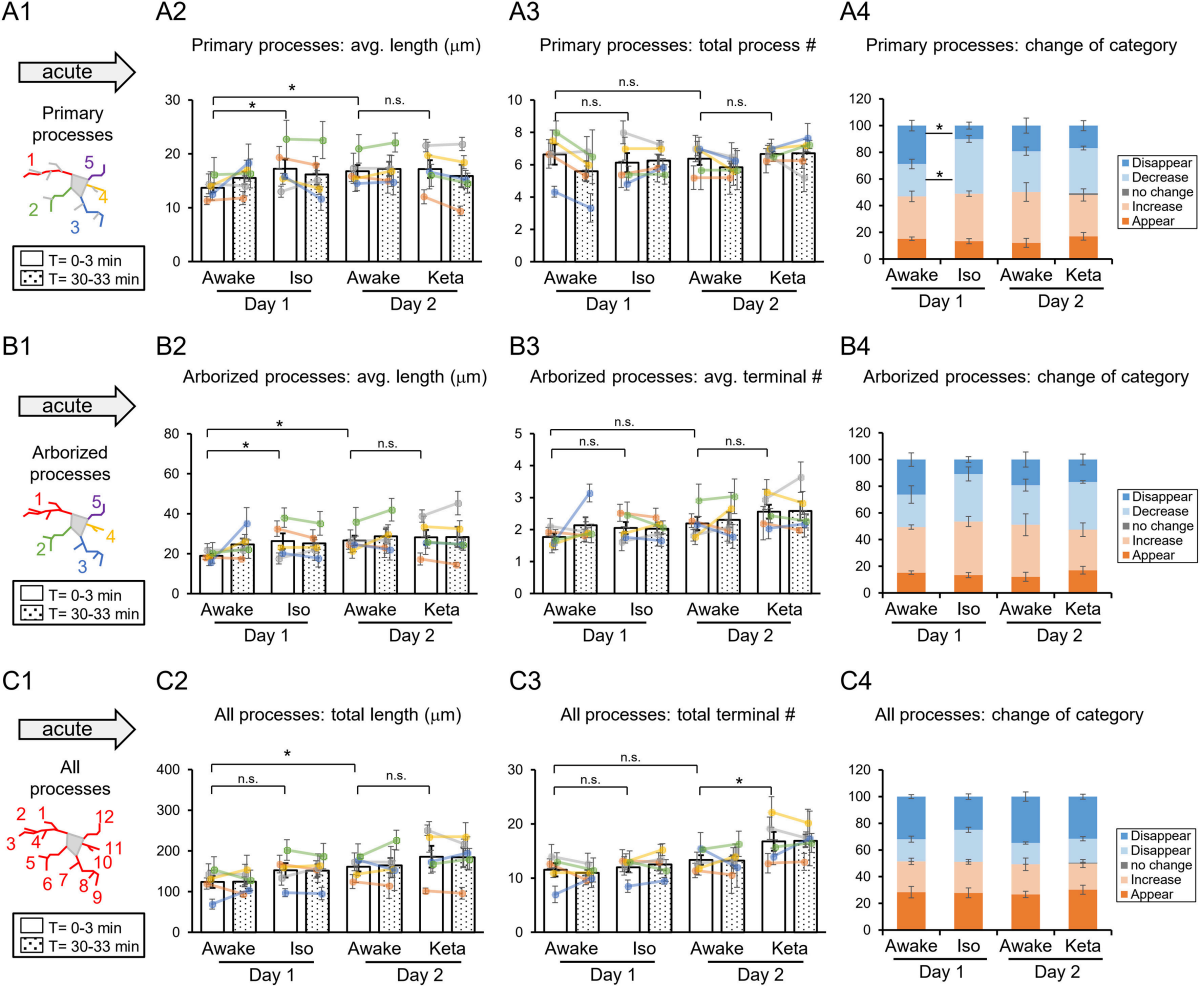
Dynamics of microglial processes in awake, isoflurane (Iso)- and ketamine (Keta)-anesthetized mice in acute experiments (5 animals). Schematic drawing of microglial processes (represented in color-matching mode): primary processes **(A1)**, arborized processes **(B1)** and all processes **(C1)**. Quantification of the average or total length and the total terminal number per primary process **(A2,A3)**, arborized process **(B2,B3)** and all processes **(C2,C3)**. The dots show individual mean values ± SEMs per animal, the values from the same animal are connected by lines **(B2,B3,C2,C3)**. White and dotted bars show means ± SEMs for all animals at time = 0 and 30 min. Processes are categorized according to morphological changes, such as “Disappear,” “Decrease,” “No change,” “Increase,” or “Appear” **(A4,B4,C4)**. ^*^p < 0.05, n.s., non-significant, one-way RM nested ANOVA, Newman-Keuls *post-hoc* test for A2–C2; Kruskal-Wallis test with Conover-Iman multiple comparison tests for A3–C3 and A4–C4.

**Figure 3:**
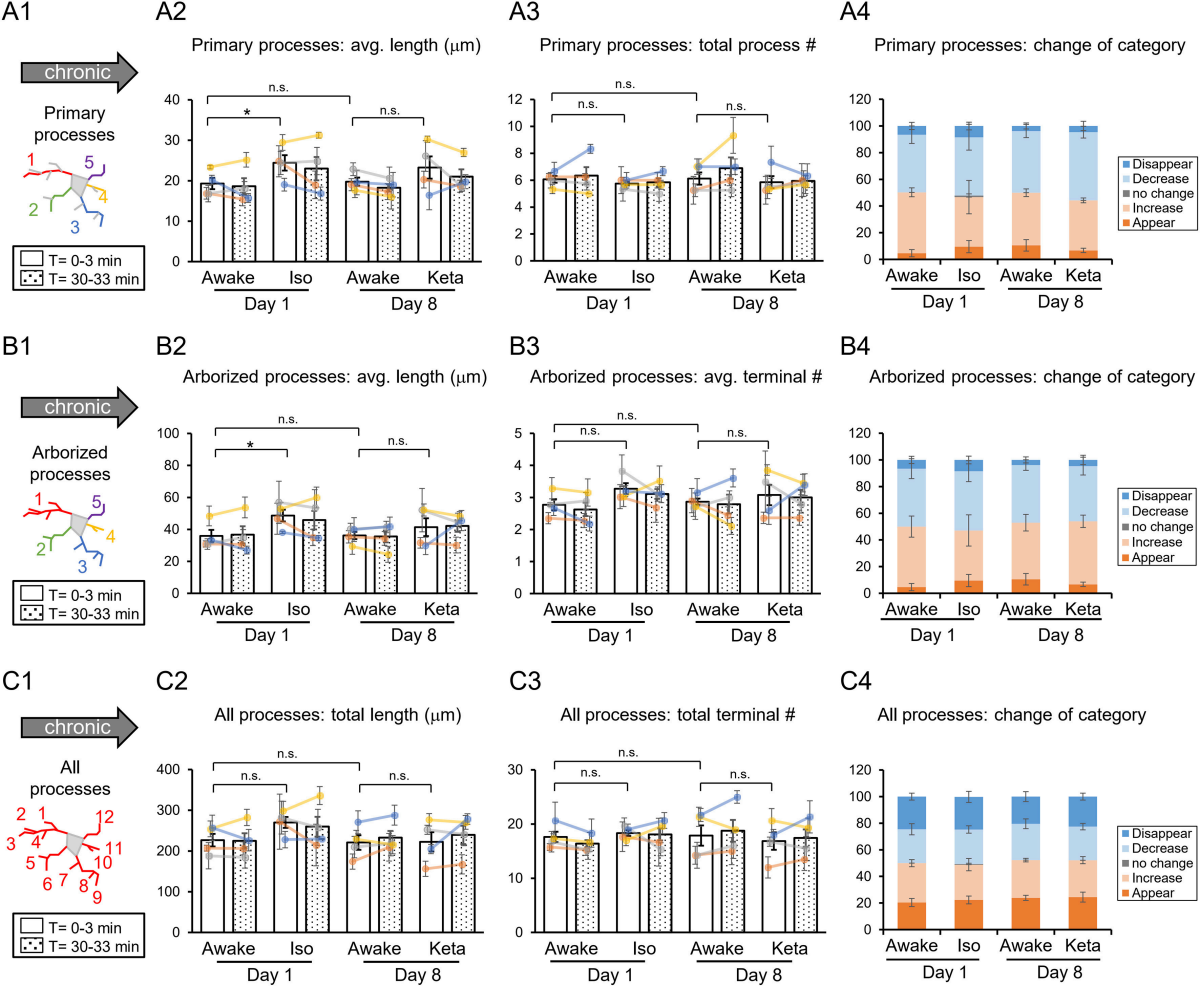
Dynamics of microglial processes in awake, isoflurane (Iso)- and ketamine (Keta)-anesthetized mice in chronic experiments (4 animals). Schematic drawings of microglial processes to be analyzed (in colors): primary processes **(A1)**, arborized processes **(B1)**, and all processes **(C1)**. Quantification of average or total length and total terminal number of primary processes **(A2,A3)**, arborized processes **(B2,B3)** and all processes **(C2,C3)**. The dots show individual mean values ± SEMs per animal, the values from the same animal are connected by lines **(B2,B3,C2,C3)**. White and dotted bars show means ± SEMs for all animals at time = 0 and 30 min. Processes are categorized according to morphological changes, such as “Disappear,” “Decrease,” “No change,” “Increase,” or “Appear” **(A4,B4,C4)**. ^*^p < 0.05, n.s., non-significant, one-way RM nested ANOVA, Newman-Keuls *post-hoc* test for A2–C2; Kruskal-Wallis test with Conover-Iman multiple comparison tests for A3–C3 and A4–C4.

To characterize microglia at the surveilling state, we traced their processes and compared the length and number of primary microglial processes (the longest processes originating from the soma, without secondary, tertiary, etc. branches; Figure 2A1), as well as the length and number of terminals of arborized processes, i.e., including secondary but not tertiary or further branches (Figure 2B1). Then, we analyzed the total length and number of terminals of all processes per cell (Figure 2C1). The same cells were traced twice, at time 0 and in 30 min (white and dotted columns, respectively, in Figures 2, 3) to quantify the cell motility. A representative movie of microglia at the surveilling state is shown in Supplementary Material.

In acute preparation, we found a difference between measurements done in awake and anesthetized conditions (using one-way repeated measures (RM) nested ANOVA for the length of processes and Kruskal-Wallis test for the number of processes and the change of the categories), with the type of anesthesia affecting the microglia processes in different ways (Table 1). During isoflurane anesthesia on day 1, microglial primary and secondary processes were significantly elongated compared to the awake condition. This was evident in terms of the average length of primary processes (*p* = 0.038; Figure 2A2) and the average length of arborized processes (*p* = 0.003; Figure 2B2). A *post-hoc* pairwise comparison showed an increase in the average length of primary and arborized processes in the isoflurane group (17.2 ± 1.7 and 26.3 ± 3.9 μm, respectively) as compared to awake mice on day 1 (13.7 ± 0.8 and 18.9 ± 1.0 μm, respectively; Figures 2A2,B2). In contrast, during ketamine anesthesia on day 2, the average length of processes was unchanged but the number of total processes was increased compared to the awake condition (total number of terminals of all processes (*p* < 0.001; Figure 2C3). Specifically, ketamine increased the total terminal number of all processes (13.4 ± 0.9 vs. 16.8 ± 1.7; Figure 2C3). Additionally, a difference between awake mice imaged on days 1 and 2 was found in terms of process lengths (primary: 13.7 ± 0.8 and 16.8 ± 1.1 μm, respectively, Figure 2A2; arborized: 18.9 ± 1.0 and 26.6 ± 2.5 μm, respectively, Figure 2B2; all processes: 123.8 ± 14.7 and 161.1 ± 11.7 μm, respectively, Figure 2C2), suggesting that anesthetized condition, as well as imaging period, differentially affect the microglia processes in acute preparation.

**Table 1:** Summary of results: “↑” and “↓” means a significant increase and decrease, respectively, *p* < 0.05.

**Motility type**		**Acute cranial window**		**Chronic cranial window**	
		**Isoflurane**	**Ketamine**	**Isoflurane**	**Ketamine**
Microglial surveilling	Length	↑		↑	
	Ramification		↑		
	Processes' disappearance	↓			
Damage- directed	Velocity			↑	
	Activation area				

When we followed microglia processes over a 30-min interval, we did not find any differences in the process length between awake and anesthetized conditions (Supplementary Figure 1). However, a closer inspection of process dynamics revealed that the isoflurane anesthesia affected the turnover of microglia processes, with fewer primary processes disappearing and more primary processes decreasing under isoflurane anesthesia than in awake mice on day 1, while the appearance and elongation of new processes were not affected (Figures 2A4–C4).

In chronic preparation, we again found a significant difference between the awake and isoflurane-anesthetized conditions in terms of the average length of primary processes (*p* = 0.008; Figure 3A2) and the average length of arborized processes (*p* = 0.039; Figure 3B2; one-way RM nested ANOVA). In line with acute experiments, we observed an increase in the length of primary and arborized processes in isoflurane-anesthetized (24.4 ± 2.1 and 48.6 ± 4.1 μm, respectively) vs. awake conditions (19.3 ± 1.6 and 35.9 ± 4.2 μm, respectively) in chronic experiments (Figures 3A2,B2). No difference in other structural parameters and motility was detected (Figure 3).

**Figure 4:**
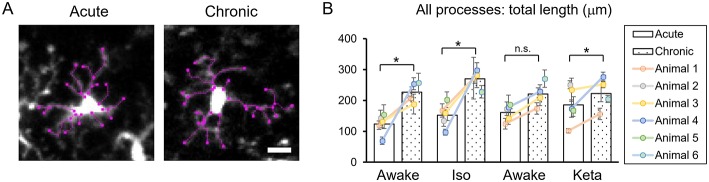
Dynamics of microglial processes in acute vs. chronic preparations. **(A)** Representative images of microglia from acute and chronic preparations, respectively (scale bar, 10 μm). **(B)** Comparison of total length of all microglial process between acute and chronic preparation in all conditions. The dots show individual mean values ± SEMs per animal, the values from the same animal are connected by lines. White and dotted bars show means ± SEMs for all animals in acute and chronic preparations. ^*^p < 0.05, n.s., non-significant, the Newman-Keuls *post-hoc* test.

Analysis of acute vs. chronic preparation revealed the effect of an interaction between Condition and Preparation on the total length of all processes (*p* = 0.017; Supplementary Table 2). We observed an increase in the total length in chronic preparation (226.7 ± 17.2 μm) vs. acute preparation in the first awake condition (123.8 ± 14.7 μm; Figure 4), suggesting that in the chronic preparation microglia are more in the surveilling rather than activated state in contrast to the acute preparation. Statistical summary for comparisons of parameters at the resting state is displayed in Supplementary Table 1.

### Analysis of Damage-Directed Microglia Response

**Figure 5:**
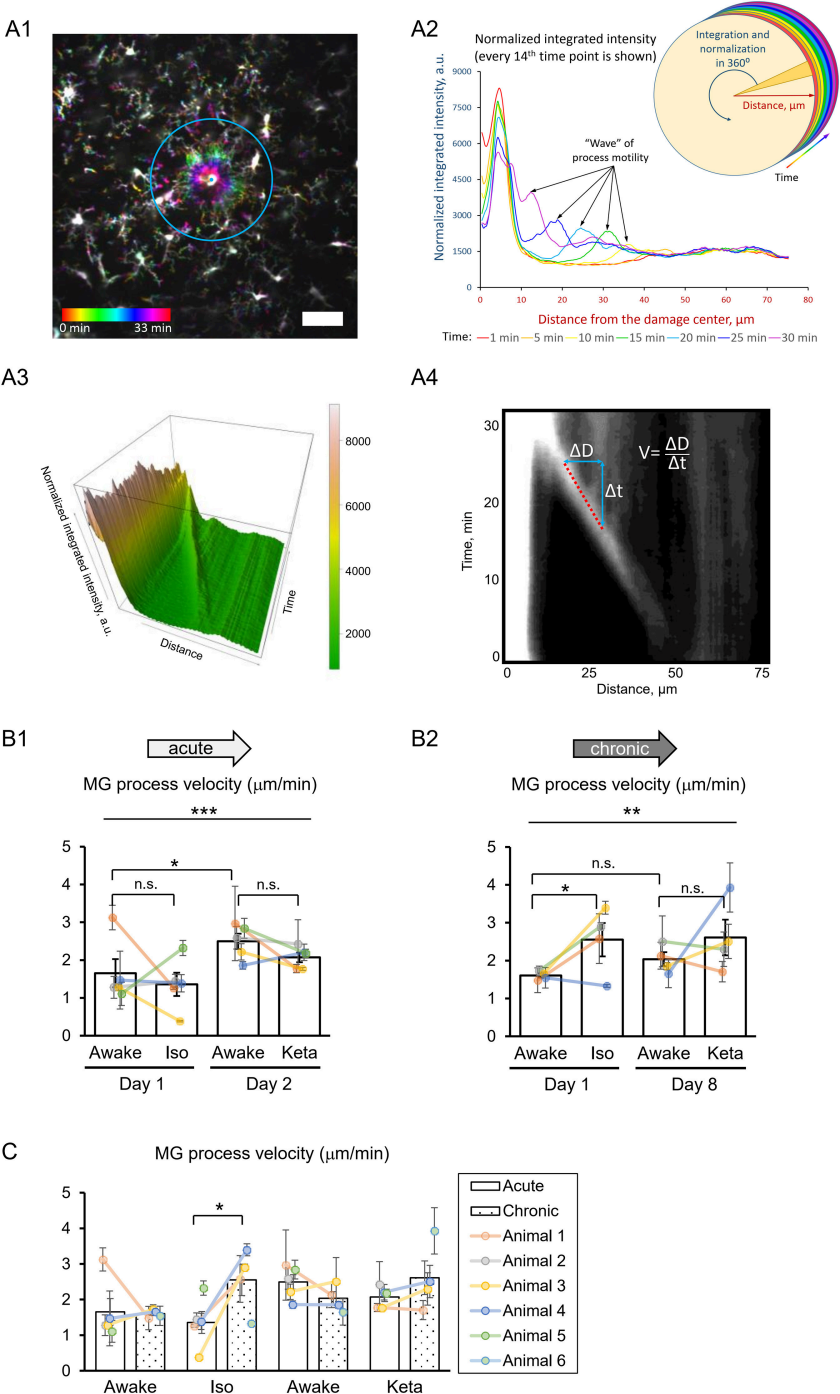
3D analysis of the photodamage-directed response of microglia. **(A1)** In order to get a better visualization of the events happening during the microglia activity, a time stack is converted into a color-coded image using a standard Fiji (ImageJ) function, where each time point is coded with its own color (scale bar, 50 μm). **(A2)** Sholl-analysis of fluorescence intensity as a function of distance from the damage site with colors matching those in **(A1)**. **(A3)** The three-dimensional plot to show the advance of microglial processes to the damage site. **(A4)** The same data as in **(A3)** are shown as a gray-coded image, with a line used to calculate the velocity. B. Quantification of microglial process velocity in acute **(B1)** and chronic **(B2)** experiments. **(C)** Comparison of microglial process velocity between acute and chronic experiments. The dots show individual mean values ± SEMs per animal, the values from the same animal are connected by lines (**B1,B2,C**). Bars show means ± SEMs values for all animals. ^*^p < 0.05, ^**^p < 0.01, ^***^p < 0.001, n.s., non-significant, the Newman-Keuls *post-hoc* test, 5 animals for acute and 4 animals for chronic experiments.

To study the damage-directed microglia response, we induced photoablation of a single microglial cell. Laser irradiation of microglia soma resulted in a collapse of the microglia processes. The site of damage remained visible as a fluorescent spot to which other microglial cells extended their processes, forming a ring-like structure (Figure 5A1). A representative movie of microglial response to a photodamage is shown in Supplementary Material. Measuring the intensity of fluorescence as a function of distance to the damage center in the time series of 3D images (Figures 5A1–3), revealed a peak (at the position of the ring) that was approaching the damage site with a constant velocity. One-way RM nested ANOVA revealed a difference between four conditions in terms of velocity in both acute and chronic preparations (*p* = 0.001 and *p* = 0.005, respectively; Figures 5B1, 2). In acute experiments, there was no effect of anesthetics, but there was a significant difference between days 1 and 2 under awake conditions (1.7 ± 0.4 and 2.5 ± 0.2 μm/min, respectively). In chronic experiments, the velocity was increased under isoflurane anesthesia (2.6 ± 0.4 μm/min) compared to awake condition (1.6 ± 0.1 μm/min) on day 1. Direct comparison between acute and chronic preparation revealed that a faster process velocity was seen in isoflurane condition in chronic than in acute preparations (Figure 5C), suggesting that the isoflurane differently affect microglia response in these experimental conditions. A slightly different method for measurements of velocity was based on the determination (for each frame) of the distance where the maximal fluorescence intensity was observed (Supplementary Figures 3A1,A2). Then a linear regression was used to determine the velocity (Supplementary Figure 3A3). Noteworthy, the linear regression provided an excellent fit to data (*R*^2^ > 0.9) and the results obtained by this method (Supplementary Figures 3B1–2, C1–2) perfectly matched the outcome of the aforementioned analysis of velocity.

Additional analysis of damage-directed microglia behavior by measuring the cumulative intensity of microglial processes in the area surrounding the photodamage site revealed that at some sites there was a biphasic response: immediately after photodamage, microglia first moved away from the damage, i.e., was repelled from, and then attracted to the damage site (Supplementary Figure 2). Hence, as another measure of microglial activation, we estimated the maximal size of the area surrounded by microglia processes in a ring-like manner at the moment when the frontier of microglia processes starts to advance toward the damage site. There was large variability in this activation area in acute experiments (*p* = 0.006; Supplementary Figure 4A). On average, the profile of the mean activation area in all experimental groups mirrored that of mean velocity (Figure 5B). Correlation analysis of velocity and activation area within individual experiments revealed a highly significant correlation between these parameters both in acute experiments (Spearman *r* = −0.576, *p* = 0.00014), and in chronic experiments (Spearman *r* = −0.371, *p* = 0.043), particularly under isoflurane anesthesia (Spearman *r* = −0.526, *p* = 0.030). To find out whether the velocity also depended on other parameters in photodamage experiments, we performed correlation analysis for the damage area, estimated as the fluorescent area at the site of laser irradiation, the number of microglia cells adjacent to the damage site and the mean distance between their somata and the damage site. There were no significant correlations between these parameters and velocity in acute and chronic experiments (Supplementary Figure 4). Statistical summary for comparisons of parameters in response to a photodamage is displayed in Supplementary Table 3.

Taken together, these data demonstrate that morphology and motility of microglia *in vivo* are strongly affected by such experimental conditions as the preparation type (acute vs. chronic preparation) and state of consciousness (awake vs. ketamine-anesthetized vs. isoflurane-anesthetized).

## Discussion

The main findings of this study are the significant effects of both the preparation type and the anesthetics on microglial morphology, motility, and response to damage. Notably, the potentiating effect of isoflurane on microglial motility that we observed using *in vivo* imaging is in sharp constant with the motility-suppressing effect of isoflurane observed previously using *ex vivo* and *in vitro* imaging (Madry et al., 2018), once again highlighting the need for caution when extrapolating the results of *ex vivo*/*in vitro* experiments to *in vivo* conditions. However, we acknowledge that the effect of anesthesia depends on the readout used for analysis.

In the chronic preparation, where the cranial window was implanted 4 months prior to imaging, isoflurane (but not ketamine) significantly increased the length of microglial processes and accelerated their damage-directed motility. These effects are consistent with the notion that isoflurane enhances the homeostatic microglial activity and reinforces the “resting-state” phenotype of microglia characterized by highly-ramified morphology and rapid reaction to microscale lesions. Ketamine anesthesia had no effect on the parameters quantified in this study (Table 1), suggesting that this injectable anesthetic may be more suitable than gaseous ones (such as isoflurane) for chronic microglia imaging.

In acute preparation, isoflurane also induced significant elongation of microglial processes like in chronic preparation; however, isoflurane did not accelerate damage-directed microglial motility (Table 1). It seems plausible to suggest that in the acute preparation the strong effects of post-surgical inflammation on microglial motility and morphology may have masked the effect of isoflurane on damage-directed motility. Ketamine significantly increased microglial ramification under acute conditions, in contrast to chronic conditions where ketamine had no effect (Table 1). These findings indicate that putative post-surgical inflammation occurring during the first days after acute window implantation can have a dramatic effect on microglial motility and morphology, comparable to or even more prominent than the effects of anesthesia.

Interestingly, certain time-dependent changes observed in the acute preparation apparently resulted in a variety of alterations in microglial morphology and dynamics. Indeed, compared to post-surgical day 1, on day 2 the microglial processes were significantly elongated (Figures 2A2–C2), the damage-directed motility was significantly accelerated (Figure 5B1), and the activation area surrounding the damaged site was reduced (Supplementary Figure 3B2). While elucidation of the mechanisms underlying the observed time-dependent changes will require further studies, these changes are most likely related to a gradual onset of post-surgical inflammation.

Our findings, therefore, highlight the need for caution in interpreting data obtained with acute cranial windows (i.e., windows freshly prepared on the same day or 1–2 days prior to imaging). In sharp contrast to acute preparation, in chronic cranial windows, none of the quantified microglial parameters changed significantly between first and second imaging sessions (Figures 3A3–C3, Figure 5B2, and Supplementary Figure 3C). This is consistent with a relative stability of microglial morpho-functional parameters in chronic preparation, and is also in line with the conclusion of a previous study (Dorand et al., 2014) that a resting period of at least 1–3 weeks is preferable before collecting experimental data on microglia in order to minimize local and systemic host responses toward potential damage resulting from the surgical procedure. As a valuable alternative preparation, a thinned-skull window has been widely used for imaging microglia *in vivo*, both for acute experiments (Wake et al., 2009; Liu et al., 2010) and chronic experiments (after implanting a coverslip on top of the thinned bone: Marker et al., 2010; Yang et al., 2010). This type of preparation is believed to minimize the inflammatory background unavoidable in cranial window preparations. However, the thinned-skull approach has limitations for deep imaging studies, and studies requiring a large visual field or direct access to brain parenchyma for drug or virus delivery. Therefore, our results outline optimal conditions for acute and chronic imaging in applications that may benefit from cranial window preparation due to a necessity to perform deep high-resolution imaging, for instance of interaction between microglia processes and presynaptic boutons or postsynaptic spines, or reproducible, stereotypical induction of the laser injury with a fixed set of parameters.

An alternative explanation for significant alterations in microglia dynamics observed on day 2 in acute preparation is that the effect of isoflurane administered on day 1 did not subside completely by the time when the same animal was re-imaged in awake conditions (day 2). A recent study has demonstrated that isoflurane anesthesia promoted phosphorylation of TrkB receptors in several brain regions (Antila et al., 2017). Although the phosphorylation *per se* was transient and subsided in 15 min (Antila et al., 2017), the downstream effects of TrkB phosphorylation may be much more long-lasting, as reported for clinical effects of isoflurane (Langer et al., 1985, 1995; Weeks et al., 2013). With the present design of the experiment, we intended to avoid possible long-lasting effects of ketamine anesthesia done on day 1 on subsequent imaging on day 2. However, we acknowledge that this approach imposes some limitations in the interpretation of our data due to the possible effect of previous exposures to anesthesia and/or laser injuries may affect the readout on the subsequent imaging day.

There are multiple mechanisms by which anesthetics (both volatile and injectable) could affect microglial dynamics. One of ketamine's best-characterized mechanisms of action is via inhibition of NMDA-type glutamate receptors, which are also expressed by microglial cells (Kaindl et al., 2008; Murugan et al., 2013). Furthermore, ketamine can exert its action via other receptors and/or signaling pathways, including brain-derived neurotrophic factor, which is considered to be a key player in ketamine's antidepressant action (Björkholm and Monteggia, 2016) as well as TLR3, NO and HCN1 (Mei et al., 2011; Sleigh et al., 2014). Considering ketamine's well-documented potentiating effect on dendritic spine formation rate (Li et al., 2010; Phoumthipphavong et al., 2016; Pryazhnikov et al., 2018), and the role of activated microglia in spine elimination under pathological conditions (Bisht et al., 2016, 2018), it is reasonable to hypothesize that ketamine's spine-promoting effect may, at least in part, be due to ketamine suppressing microglial activation. Indeed, our results demonstrate that, at least in acute preparation, ketamine increased ramification of microglial processes. The fact that this effect was not seen in chronic conditions suggests that the influence of ketamine on microglia is enhanced by a global stressor (i.e., surgery), while local damage did not result in a detectable influence of ketamine.

Like ketamine, isoflurane has been reported to affect microglia via several distinct mechanisms, involving modulation of NMDA receptors (which are inhibited by isoflurane, albeit less potently than by ketamine) as well as the TLR4/NF-kB pathway and GABAA receptors (Brosnan, 2011; Xiang et al., 2014; Sun et al., 2015). Changes in microglia behavior under isoflurane anesthesia are noteworthy in the context of the isoflurane-induced elevation in TrkB signaling (Antila et al., 2017) that might reflect the microglial release of BDNF or matrix metalloproteinases converting pro-BDNF to mature BDNF. In a recent study (Szalay et al., 2016), longitudinal two-photon imaging was performed in isoflurane-anesthetized mice to demonstrate a protective role of microglia in a model of stroke. While the authors argue that the low-level isoflurane (0.8–1.1%) used during imaging should not influence the observed detrimental effect of microglia removal on neuronal activity, it is noteworthy that a slightly higher level of isoflurane (1.5%) used during imaging in the current study, did have a significant effect on microglial process morphology, in both acute and chronic preparations (see Table 1).

In conclusion, considering the reproducibility and quality of data obtained with two-photon imaging of microglia in awake mice with a chronically implanted transcranial window, we recommend this approach for the studies of microglia morphology and function *in vivo* in adult animals. Obviously, this approach allows to avoid the confounding effects of anesthetics and transient inflammation induced by window implantation. Because we did not detect any differences between awake and ketamine-anesthetized mice with a chronically implanted transcranial window, the ketamine anesthesia could be a useful alternative to awake imaging, at least for analysis of parameters quantified in the present studies. However, other experimental factors, such as mouse genotype or global experimental stressors, may interact with chronic inflammation and ketamine in a yet unknown fashion and unpredictably bias the outcome; thus, microscopic imaging experiments in awake mice still should be preferred. Apart from the Mobile Home Cage used in the current study, alternative methods have been employed for imaging of head-fixed awake mice (Rangroo Thrane et al., 2012; Akiyoshi et al., 2018). If a laboratory is not equipped with a head-fixation device that allows awake imaging, then ketamine anesthesia could be preferable to isoflurane.

## Ethics Statement

All animals were treated in strict accordance with ethical animal research standards defined by the German law and approved by the Ethical Committee on Animal Health and Care of Saxony-Anhalt state, Germany (license number: 42502-2-1346).

## Author Contributions

AD and LK initiated the study, SS and WS generated a detailed plan of experiments. SS, AD, LK, and MY supervised the research. WS performed imaging experiments, KS, DT, and AD analyzed the data. AD, LK, WS, and KS wrote the first draft. All authors edited the manuscript.

### Conflict of Interest Statement

LK is a co-founder and CSO of Neurotar Ltd that produces the Mobile HomeCage devices; DT is an employee of Neurotar Ltd. The remaining authors declare that the research was conducted in the absence of any commercial or financial relationships that could be construed as a potential conflict of interest.

## Supplementary Material

Supplementary Video 1 Representative movie of microglia at the surveilling state.

Supplementary Video 2 Representative movie of microglial response to a photodamage.
