# Supplementary material for: In vivo Two-Photon Imaging of Anesthesia-Specific Alterations in Microglial Surveillance and Photodamage-Directed Motility in Mouse Cortex
Source: Front Neurosci. 2019 May 7;13:421. doi: 10.3389/fnins.2019.00421 (PMC6513965; doi:10.3389/fnins.2019.00421)
Supplement: Supplementary file 1 [file Table_1.pdf]

Dynamics (Figs. 2 and 3)			Type III ANOVA p-value		significance by post hoc Newman-Keuls		
Preperation	Process	Parameter	condition	condition*animal	awake vs iso	awake vs awake 2	awake 2 vs keta
Acute (T=0)	primary	tot length	0.099	0.180	no	no	no
		avg length	**0.038**	**0.039**	**yes**	**yes**	no
		tot #	0.524	**0.030**	no	no	no
	arborized	avg length	**0.003**	**0.002**	**yes**	**yes**	no
		avg #	**0.002**	**0.034**	no	no	no
	all	tot length	**0.002**	**0.003**	no	**yes**	no
		tot #	**0.000**	**0.036**	no	no	**yes**
Chronic (T=0)	primary	tot length	0.160	0.223	no	no	no
		avg length	**0.008**	**0.035**	**yes**	no	no
		tot #	0.888	0.713	no	no	no
	arborized	avg length	**0.039**	0.199	**yes**	no	no
		avg #	0.358	0.399	no	no	no
	all	tot length	0.051	0.102	no	no	no
		tot #	0.708	0.124	No	no	no

Supplementary Table 1| Statistical summary for comparisons of parameters at the resting state of microglia.

Change in the length (Fig. S1)			Type III ANOVA p-value		significance by post hoc Newman-Keuls		
Preperation	Process	Parameter	condition	condition*animal	awake vs iso	awake vs awake 2	awake 2 vs keta
Acute	primary	tot length	0.921	0.206	no	no	no
		avg length	0.914	0.351	no	no	no
	arborized	avg length	0.913	0.223	no	no	no
	all	tot length	0.985	0.287	no	no	no
Chronic	primary	tot length	0.699	0.066	no	no	no
		avg length	0.686	0.136	no	no	no
	arborized	avg length	0.744	0.095	no	no	no
	all	tot length	0.294	**0.012**	no	no	no

Category (Figs.2&3-A4, B4 and C4)			Type III ANOVA p-value		significance by post hoc Newman-Keuls		
Preperation	Process		condition	condition*category	awake vs iso	awake vs awake2	awake2 vs keta
Acute	primary			**0.001**	**decrease, disappear**	no	no
	arborized			**0.033**	no	no	no
	all			**0.008**	no	no	no
Chronic	primary			0.813	no	no	no
	arborized			0.980	no	no	no
	all			0.959	no	no	no

acute *versus* chronic (Fig. 4)			Type III ANOVA p-value		significance by post hoc Newman-Keuls			
Preperation	Process	Parameter	preparation	condition*preparation	awake	iso	awake2	keta
acute *vs* chronic (T=0)	primary	tot length	**< 0.0001**	0.119	**yes**	**yes**	no	**yes**
		avg length	**< 0.0001**	0.094	**yes**	**yes**	no	**yes**
		tot #	**< 0.0001**	0.240	no	no	no	no
	arborized	avg length	**< 0.0001**	0.142	**yes**	**yes**	no	**yes**
		avg #	**< 0.0001**	0.880	**yes**	no	no	**yes**
	all	tot length	**< 0.0001**	**0.017**	**yes**	**yes**	no	**yes**
		tot #	**< 0.0001**	0.463	**yes**	**yes**	**yes**	no

Supplementary Table 2| Statistical summary for comparisons of parameters between acute and chronic preparations at the resting state of microglia.

Velocity (Fig. 5B)		Type III ANOVA p-value		significance by post hoc Newman-Keuls		
Preperation	Parameter	condition	condition*animal	awake vs iso	awake vs awake2	awake 2 vs keta
Acute	velocity	**0.001**	**0.034**	no	**yes**	no
Chronic	velocity	**0.005**	**0.009**	**yes**	no	yes

acute *versus* chronic (Fig. 5C)		Type III ANOVA p-value		significance by post hoc Newman-Keuls			
Preperation	Parameter	condition	condition*preparation	awake	iso	awake2	keta
acute *vs* chronic	velocity	**< 0.0001**	**0.000**	no	**yes**	no	no

Supplementary Table 3| Statistical summary for comparisons of microglia parameters in response to a photodamage.
