# Supplementary material for: In vivo Two-Photon Imaging of Anesthesia-Specific Alterations in Microglial Surveillance and Photodamage-Directed Motility in Mouse Cortex
Source: Front Neurosci. 2019 May 7;13:421. doi: 10.3389/fnins.2019.00421 (PMC6513965; doi:10.3389/fnins.2019.00421)
Supplement: Supplementary file 4 [file Presentation_1.PPTX]

## Slide 1
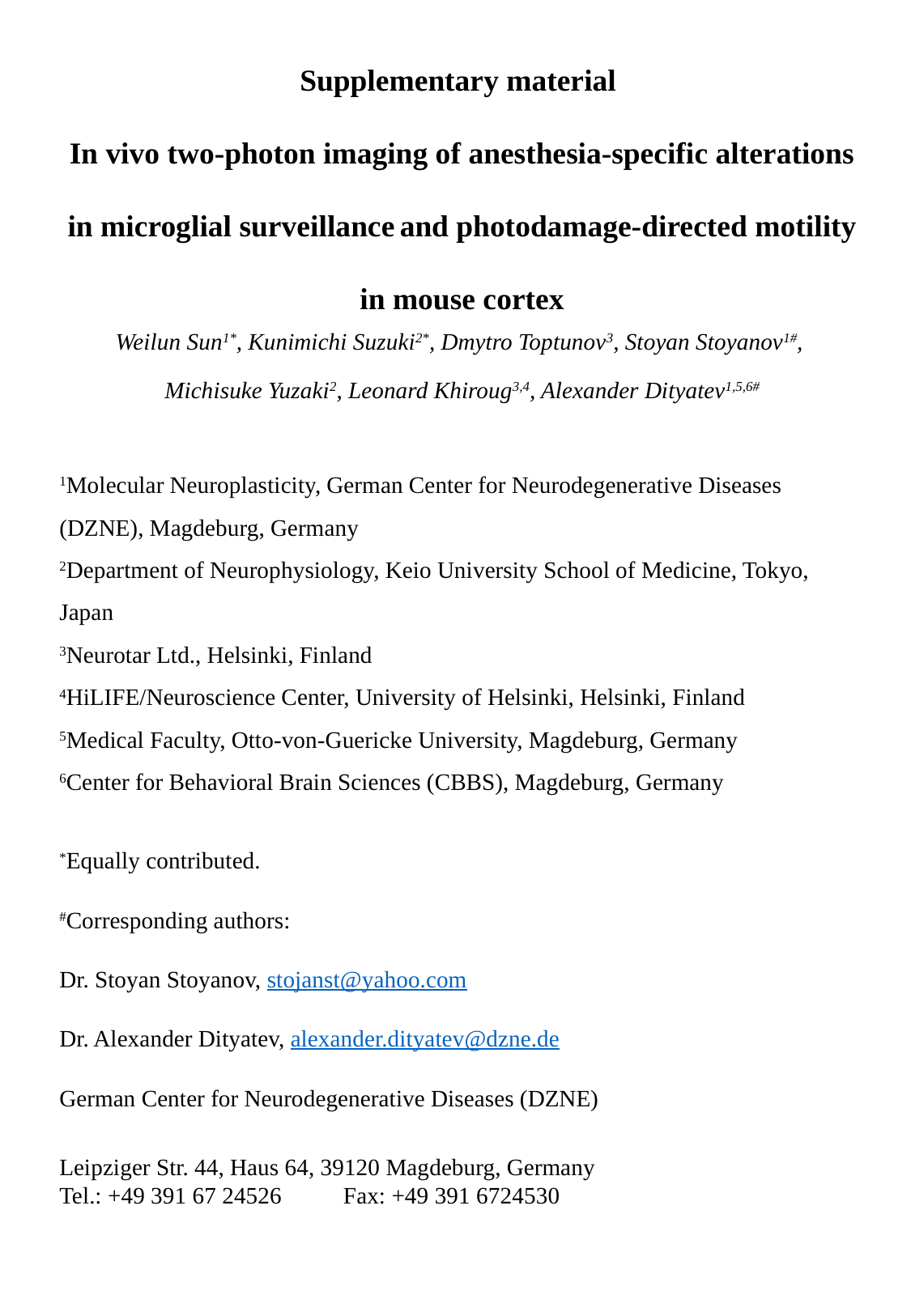

Supplementary material
In vivo two-photon imaging of anesthesia-specific alterations in microglial surveillance and photodamage-directed motility in mouse cortex
Weilun Sun1*, Kunimichi Suzuki2*, Dmytro Toptunov3, Stoyan Stoyanov1#,
Michisuke Yuzaki2, Leonard Khiroug3,4, Alexander Dityatev1,5,6#
1Molecular Neuroplasticity, German Center for Neurodegenerative Diseases (DZNE), Magdeburg, Germany
2Department of Neurophysiology, Keio University School of Medicine, Tokyo, Japan
3Neurotar Ltd., Helsinki, Finland
4HiLIFE/Neuroscience Center, University of Helsinki, Helsinki, Finland
5Medical Faculty, Otto-von-Guericke University, Magdeburg, Germany
6Center for Behavioral Brain Sciences (CBBS), Magdeburg, Germany
*Equally contributed.
#Corresponding authors:
Dr. Stoyan Stoyanov, stojanst@yahoo.com
Dr. Alexander Dityatev, alexander.dityatev@dzne.de
German Center for Neurodegenerative Diseases (DZNE)
Leipziger Str. 44, Haus 64, 39120 Magdeburg, Germany
Tel.: +49 391 67 24526	 Fax: +49 391 6724530

## Slide 2
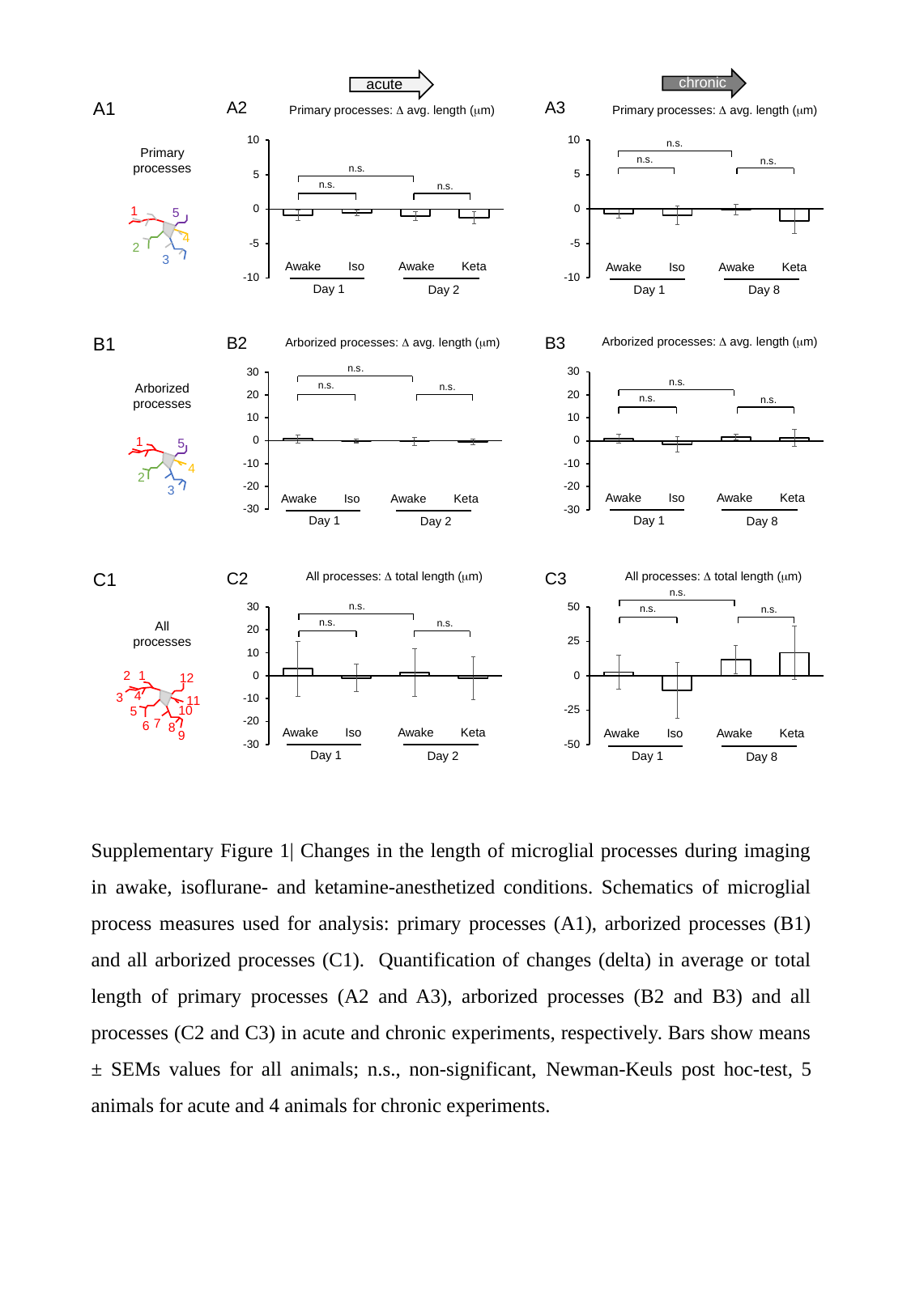

Supplementary Figure 1| Changes in the length of microglial processes during imaging in awake, isoflurane- and ketamine-anesthetized conditions. Schematics of microglial process measures used for analysis: primary processes (A1), arborized processes (B1) and all arborized processes (C1). Quantification of changes (delta) in average or total length of primary processes (A2 and A3), arborized processes (B2 and B3) and all processes (C2 and C3) in acute and chronic experiments, respectively. Bars show means ± SEMs values for all animals; n.s., non-significant, Newman-Keuls post hoc-test, 5 animals for acute and 4 animals for chronic experiments.

## Slide 3
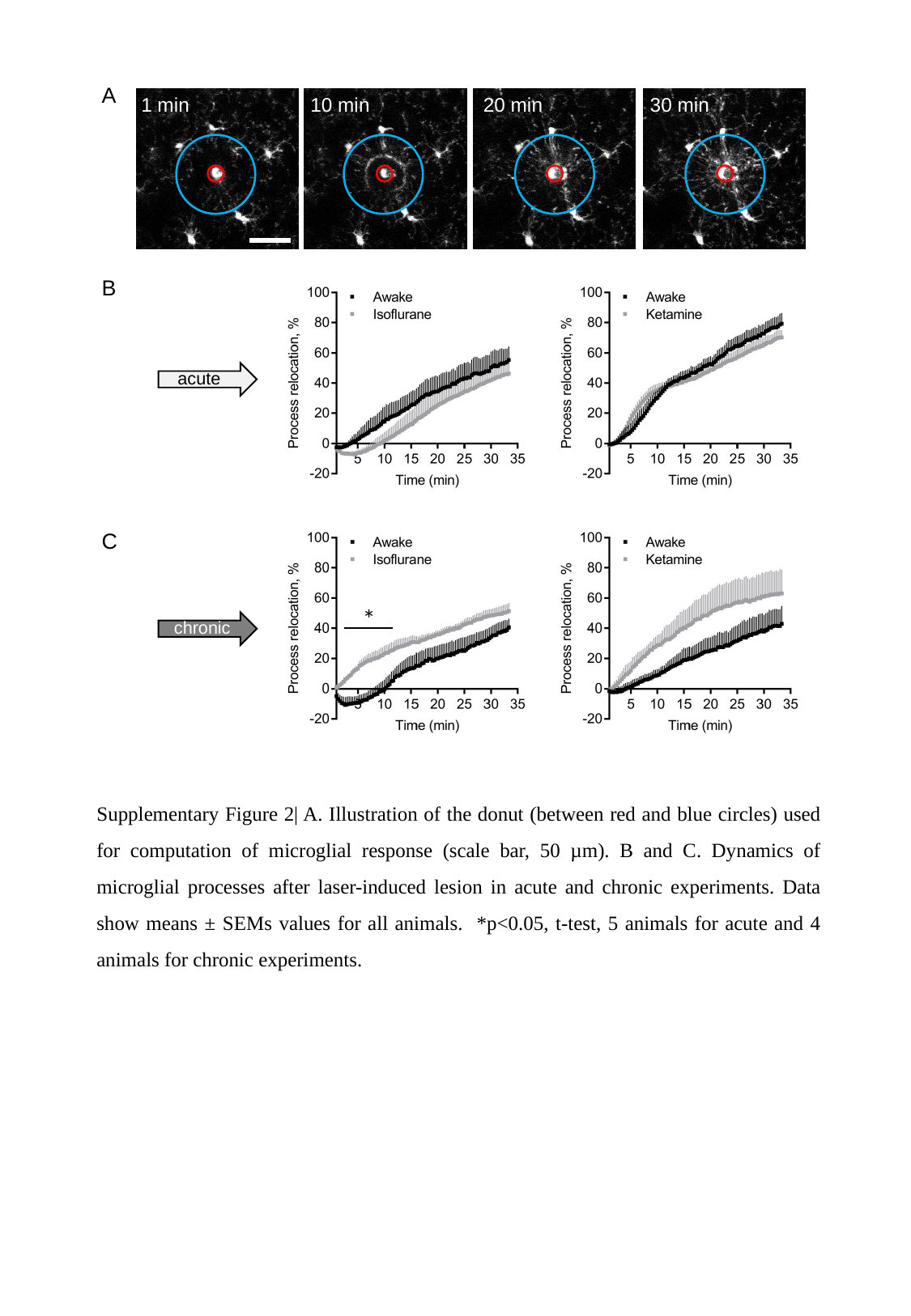

Supplementary Figure 2| A. Illustration of the donut (between red and blue circles) used for computation of microglial response (scale bar, 50 µm). B and C. Dynamics of microglial processes after laser-induced lesion in acute and chronic experiments. Data show means ± SEMs values for all animals. *p<0.05, t-test, 5 animals for acute and 4 animals for chronic experiments.

## Slide 4
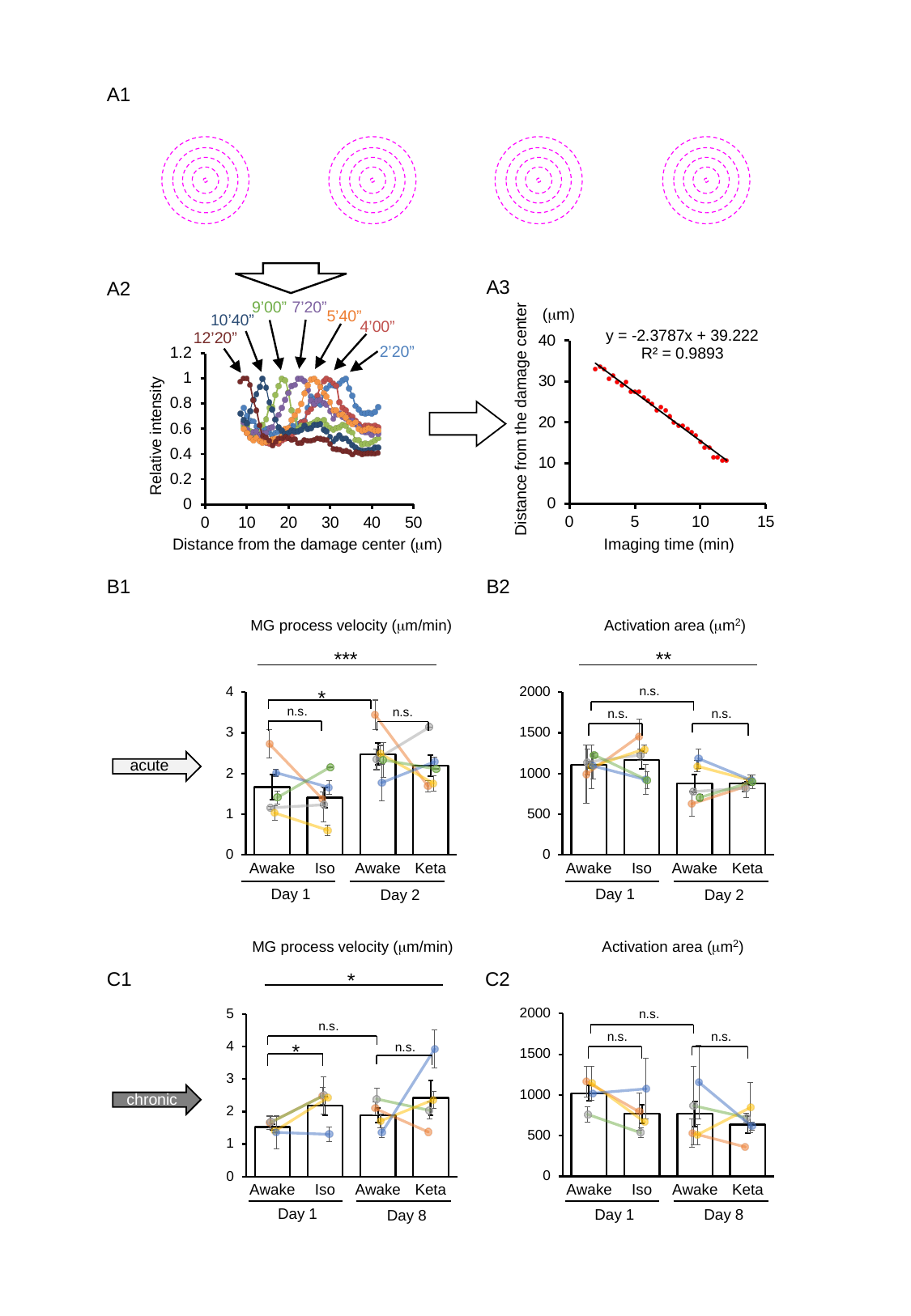

## Slide 5
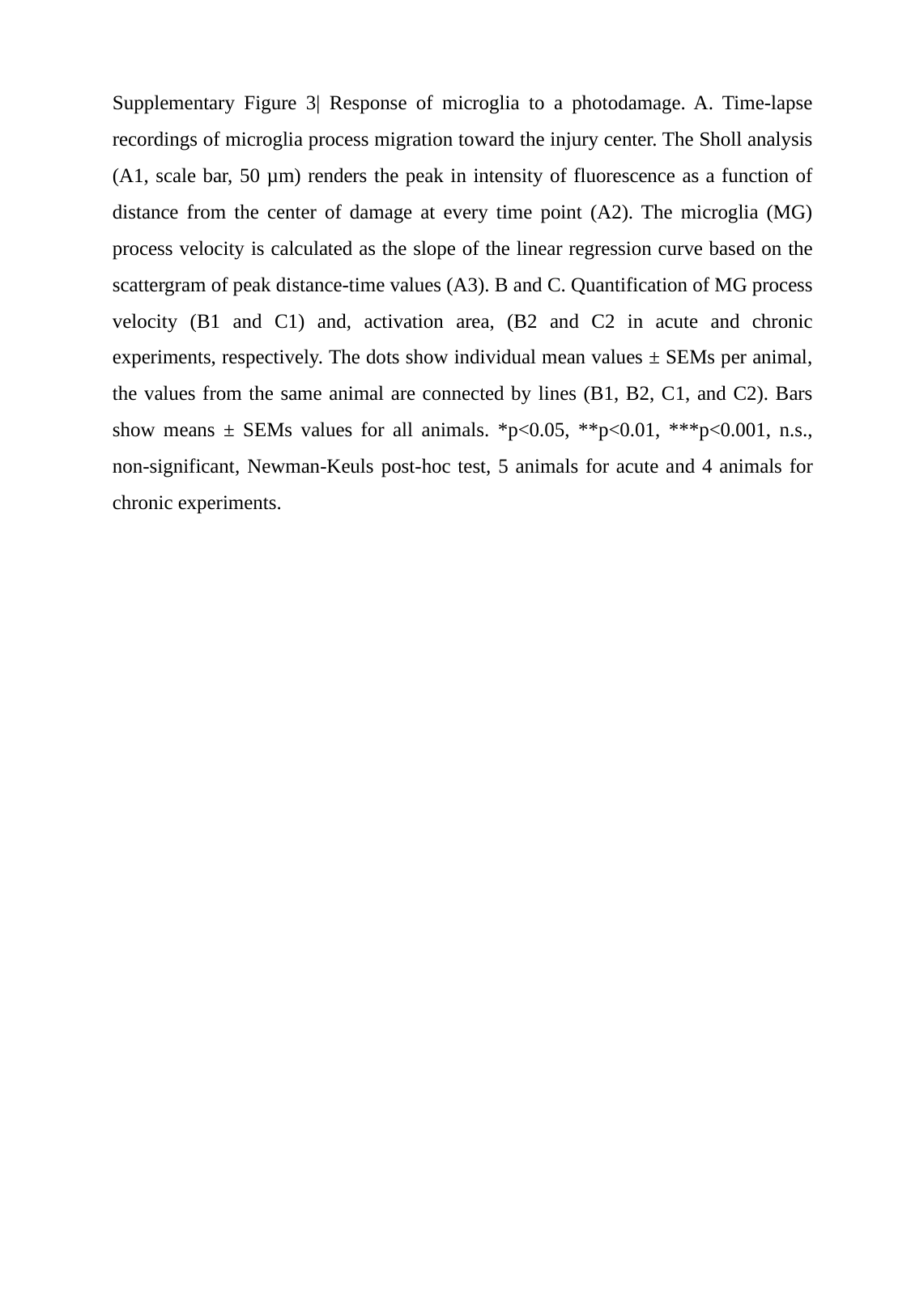

Supplementary Figure 3| Response of microglia to a photodamage. A. Time-lapse recordings of microglia process migration toward the injury center. The Sholl analysis (A1, scale bar, 50 µm) renders the peak in intensity of fluorescence as a function of distance from the center of damage at every time point (A2). The microglia (MG) process velocity is calculated as the slope of the linear regression curve based on the scattergram of peak distance-time values (A3). B and C. Quantification of MG process velocity (B1 and C1) and, activation area, (B2 and C2 in acute and chronic experiments, respectively. The dots show individual mean values ± SEMs per animal, the values from the same animal are connected by lines (B1, B2, C1, and C2). Bars show means ± SEMs values for all animals. *p<0.05, **p<0.01, ***p<0.001, n.s., non-significant, Newman-Keuls post-hoc test, 5 animals for acute and 4 animals for chronic experiments.

## Slide 6
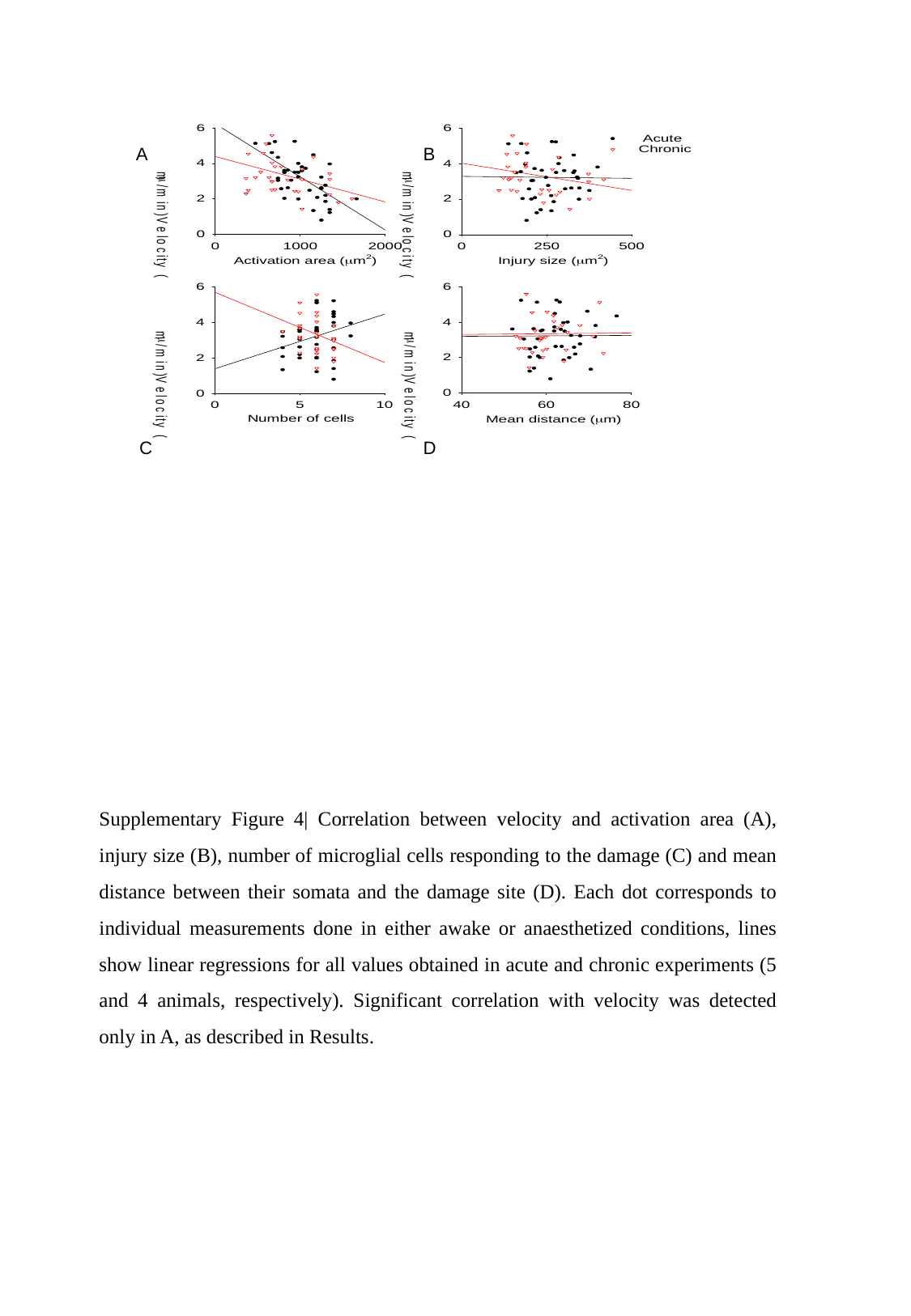

Supplementary Figure 4| Correlation between velocity and activation area (A), injury size (B), number of microglial cells responding to the damage (C) and mean distance between their somata and the damage site (D). Each dot corresponds to individual measurements done in either awake or anaesthetized conditions, lines show linear regressions for all values obtained in acute and chronic experiments (5 and 4 animals, respectively). Significant correlation with velocity was detected only in A, as described in Results.
